# Intraspecific variation in the functional responses of an invasive tropical freshwater fish under increasing temperature regimes

**DOI:** 10.1038/s41598-024-79957-0

**Published:** 2024-11-18

**Authors:** Matteo Ventura, Simone Cittadino, Edoardo Calizza, Giulio Careddu, Simona Sporta Caputi, Loreto Rossi, Maria Letizia Costantini

**Affiliations:** 1 Department of Environmental Biology, Sapienza University of Rome, Rome, 00185 Italy; 2 CoNISMa, National Inter-University Consortium for Marine Sciences, Rome, 00196 Italy

**Keywords:** Global warming, Alien species, Predator-prey interactions, Prey body size, Comparative functional response (CFR), Feeding preferences, Ecology, Climate-change ecology, Freshwater ecology, Invasive species

## Abstract

Global warming and the introduction of non-native fish represent major threats to freshwater biodiversity worldwide, but their effects have usually been investigated separately. Since most fish are ectotherms, their metabolism and feeding behaviour are highly influenced by temperature. Increasing water temperatures may thus exacerbate the impact of non-native fish, particularly those adapted to warmer conditions, on prey populations. Increasing temperature can also result in divergences between the impacts of females and males, especially in sexually dimorphic species.

The globally invasive tropical guppy *Poecilia reticulata* Peters, a popular aquarium fish also used for control of mosquito-borne diseases and as a model species in ecological and evolutionary studies, exhibits strong sexual dimorphism and larvivory. This laboratory study examined prey consumption and prey size selection by guppies fed with chironomid larvae under varying temperature conditions. The effect of sex, pregnancy and prey body size on the guppy’s predatory response was also assessed by comparing Functional Responses.

The results highlighted four key points: (1) increased temperature led to increased prey consumption in both females and males by decreasing handling time; (2) prey consumption was disproportionately higher in females than males, regardless of temperature; (3) temperature influenced females’ prey size selection; and (4) pregnancy reduced prey handling time among females.

These findings show that temperature and intraspecific differences influence the feeding response of invasive fish, and they should both be taken into account when investigating and predicting the ecological impact of invasive species on invaded food webs.

## Introduction

Increasing temperatures and biological invasions pose major threats to biodiversity and ecosystem functioning worldwide^1–3^. They particularly affect freshwater ecosystems, where temperatures are expected to increase by up to 6 °C by the end of this century^4–8^, and introductions of alien species are increasingly frequent^9–11^.

Freshwater ecosystems host at least 9.5% of the Earth’s animal biodiversity, including one-third of global vertebrate species and over 10,000 fish species (approximately 40% of global fish diversity^12–14^). However, freshwater ecosystems are often isolated or fragmented, representing ecological islands for aquatic species^15–17^. This means that dispersal is limited and the capacity of native aquatic species to cope with environmental change is thus reduced^16,18–20^. Therefore, as a consequence of ongoing global changes, freshwater species are declining more rapidly than marine or terrestrial ones^21,22^, with the introduction of alien species seen as a major factor^23–25^.

Fish are among the most widely introduced aquatic animals^26^, and their impact is commonly associated with their predatory capacity^23,24,27,28^. Since most fish are ectotherms, their metabolic rate, growth and feeding activity are strongly affected by temperature^29^. Due to climate change, some introduced fish species, especially eurytherms and generalists, may experience thermal conditions that are closer to those of their original geographical range and hence more optimal. Thus, their predatory impact on freshwater resources is expected to increase worldwide^6,8,30,31^.

Given its potential to provide new insight into resilience and vulnerability to climate stressors, Gissi and colleagues^32,33^ have extensively discussed the importance of the sex-based approach to studies of climate-induced changes. Predation behaviour is a functional trait that varies depending on the phenotype. It can differ between males and females and can be relevant to the species’ responses to environmental changes including climate change. However, sex differences, which can also be observed in body size and physiology, are typically overlooked in studies of climate-driven changes and the impacts of non-native fish^32,34^. In sexually dimorphic species, females are typically larger than males^34^. Since the body size of both fish and their prey has been shown to play a key role in determining predatory interactions^35–37^, when sexual dimorphism entails differences in body size, females and males can select prey of differing size and can therefore feed on different trophic levels^37^. Moreover, since females have high energy demands for egg production, it is reasonable to hypothesise that females will have a greater impact on available prey than males, and pregnant females a greater impact than non-pregnant ones.

Although the prediction of ecological impacts is notoriously difficult, Functional Responses (FRs^38,39^), describing prey uptake in relation to density under controlled conditions, have often proved to have excellent explanatory and predictive power regarding the impact of predators on prey populations^40–43^. The essential components of FRs are the attack rate (a measure of predator efficiency at low prey densities) and handling time, which includes the time needed to capture, consume and digest prey and determines the height of the FR curve (i.e. the maximum feeding rate^44–46^).

The Comparative Functional Response (CFR) method has been used to make comparisons between native and trophically analogous invasive species^42,47^, as well as between conspecifics under a range of biotic conditions (e.g. parasitism, predation, intra and interspecific competition^42,48^) and abiotic conditions (e.g. temperature, habitat complexity, light regime^49,50^). Higher functional responses have been associated with greater impacts on prey populations in the field^42,47^. Evidence suggests that increased impact may be related to reduced handling time under warmer conditions^45,51,52^. However, differences in prey handling time due to warming have only been marginally investigated for freshwater fish^53^.

In order to determine whether rising temperature affects the feeding rate and prey size selection of invasive fish species, we measured FRs and performed feeding preference experiments based on a range of prey sizes in the larvivorous invasive guppy *Poecilia reticulata* Peters, which is also one of the most important model organisms in ecological and evolutionary studies^54^. Marked sexual dimorphism makes the guppy a highly appropriate model species, of global significance, for measuring the changing feeding response of non-native fish under climate change.

FRs were compared between males and females and between pregnancy stages in females at a range of temperatures, and prey selection was investigated. Specifically, the following four hypotheses were tested: (1) increasing temperature increases the functional response of both male and female fish; (2) the functional response of females is higher than that of males; (3) prey size selection differs between sexes and is influenced by temperature; (4) pregnancy increases females’ functional response.

## Materials and methods

### Rearing of *P. reticulata*

Native to northeastern South America, the guppy *P. reticulata* has been introduced worldwide for use in hobby aquaria and mosquito control, being recognised as invasive with negative ecological effects in several countries^55–58^. The documented impacts of the guppy, which is trophically analogous to the invasive larvivorous *Gambusia*, one of the world’s worst invasive species, include local extinction of invertebrates (e.g. *Halocaridina rubra* Holthuis in Hawaii^57^), lower densities of invertebrates and fish due to predation and competition for resources, increased primary productivity and changes in nitrogen fluxes^57,59^. It has been shown that the guppy can exhibit even higher functional responses than *Gambusia*^60^. Both species exhibit marked sexual dimorphism, with males being smaller than females^56^.

To establish an experimental fish stock comprising males and females of standardised age and body size, two pregnant female guppies belonging to the same breeding stock were reared in an aerated rearing tank with a 100 W thermostat at 25 °C and under a constant photoperiod (12-h light). The rearing tank contained 120 L of tap water treated with a water conditioner (Sera Aquatan) to neutralise chlorine residues. An internal submerged bio-filter was inserted to convert the ammonia excreted by the fish to nitrate, much less toxic. Dense synthetic vegetation was added to create shelters for incoming fry and prevent cannibalism. Regular changes of water, sourced from tap water (25% changed every week), were performed. After a few days, both females gave birth to their fry, which were isolated in aerated stock tanks under similar conditions and fed daily with specific granular food for guppies (Hikari Fancy Guppy semi-floating Granules).

As soon as the gonopodium, used for fertilisation^55^, began to develop, the males were promptly separated from the females to prevent breeding. Under similar conditions, some males and females in a ratio of 1:2 were left in the stock tanks for 48 h to obtain females at the same stage of pregnancy. The low male-to-female ratio was designed to limit stress on females, as male guppy mating behaviour is intense^55^. Males were then removed and females were monitored, pregnant females being retained for further trials. Two phases of pregnancy, specifically 19 and 26 days after male removal, were examined in order to quantify potential changes in functional response during pregnancy. After 19 days (hereafter referred to as stage 1), pregnancy was identifiable by the conspicuous emergence of the gravid spot, which was not evident in all pregnant females before then. After 26 days (hereafter referred to as stage 2), the embryos became distinguishable from the gravid spot, and the females’ abdomen showed swelling, indicating closeness to parturition.

### Experimental design and feeding trials

Rearing, acclimatisation and experimental feeding trials took place in an air-conditioned, photo- and thermally-isolated room, which allowed us to standardise photoperiod (12-h light) and minimize temperature fluctuations (± 0.2 °C) around the temperature to be tested. The water temperature in the tanks was maintained by setting the room’s air conditioner and water thermostats.

**Fig. 1:**
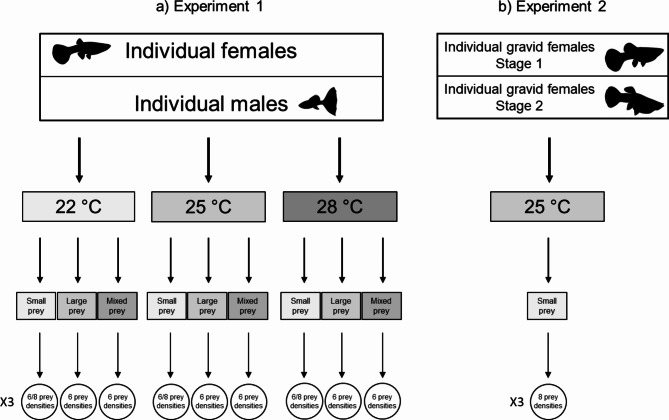
**(a)** Experimental design of the study to test the effect of sex, temperature and prey body size on the feeding response of the invasive guppy *Poecilia reticulata*. Individual female and male guppies were fed at three temperatures (22, 25 and 28 °C) with large, small and mixed-sized prey at 6 to 8 prey densities (Experiments 1). **(b)** To test the effect of pregnancy on the feeding response, gravid females (at two stages of pregnancy) were fed small prey at the medium temperature (25 °C) at 6 prey densities (Experiment 2). Each experimental condition was replicated three times. For further details, please refer to Sect. 2.2.

The experimental design included 2 experiments (Fig. 1). Experiment 1 sought to evaluate the effect of temperature and sex on (i) the guppy’s functional response to single small or large prey density and (ii) prey size selection. It included 3 replicates x 2 sexes x 3 temperatures (22, 28 and 25 °C) x 6 prey densities (2, 4, 8, 16, 32 and 64 larvae) x 3 prey treatments (single small prey, single large prey and mixed small and large prey in a 1:1 ratio). The three temperatures tested were based on the temperature range of the guppies’ original distribution (northeast South America) and the expected increase of up to 6 °C in freshwater temperatures by the end of this century^4–6,8,61^. Two additional prey densities (128 and 256) were tested in females feeding on small prey. This was necessary because the FR asymptote was not reached at the prey density of 64^62^. Prey selection was evaluated by comparing the mean number of prey consumed once the plateau was reached in the mixed and single prey-size treatments. No signs of pregnancy (i.e. the gravid spot, a darker area towards the back of the abdomen) were observed in the females of Experiment 1.

Experiment 2 sought to assess the effect of pregnancy on the females’ functional response and included 3 replicates x 2 pregnancy stages x 1 temperature (25 °C) x 8 prey densities (2, 4, 8, 16, 32, 64, 128 and 256 larvae) x 1 prey size (small prey) (Fig. 1). The effects of pregnancy were not examined at 22 and 28 °C due to the long acclimatisation period required, during which pregnancy would have ended.

Before each feeding trial, the fish in the stock tanks were acclimatised for 5 days to the temperature being tested. In order to minimise any phenotypic differences due to exposure to different conditions and/or possible body growth, the temperatures were tested in the sequence mentioned above. The transition from one temperature to another was achieved by increasing/decreasing by 1 °C per day. Twenty-four hours before each feeding trial, fish were randomly taken from the stock tanks and randomly transferred to the opaque feeding tanks measuring 19.0 cm by 13.5 cm filled with 3 L conditioned tap water. Guppies were not fed until the trial started, in order to acclimatise the single specimens to the experimental conditions and standardise hunger levels.

Individual fish were weighed to the nearest 0.01 g. Mean body weight was on average 0.91 ± 0.03 SE g and 0.33 ± 0.02 SE g for females and males respectively and did not differ between the beginning and the end of the experiments (T test, *p* always > 0.05). Pregnant females were not weighed due to the risk of miscarriage, but their age and length were similar to those of non-pregnant females.

Chironomid larvae were used as prey, as they are known to be preferred by guppies^63^. Since chironomids and guppies are both distributed globally and represent an important component of aquatic communities, they allowed us to test the effect of temperature, predator sex and prey body size while taking account of widespread interaction in freshwater food webs. Mixed sizes of *Chironomus aprilinus* Meigen larvae were purchased live (Nature Pure Bloodworms 90 ml) and separated into two size classes: 4–6 mm (mean weight ± SD: 0.13 ± 0.03 mg, hereafter “small prey”) and 10–12 mm (mean weight ± SD: 0.93 ± 0.2 mg, hereafter “large prey”).

Small and large prey were counted, divided into subgroups, and promptly frozen to standardise their quality across experiments. Before each feeding trial, the larvae were thawed and then offered to the fish. Fish were allowed to feed for 1 h and then removed from the experimental tanks. Within this time interval, defined after preliminary trials, guppies stopped feeding frantically. Uneaten larvae were collected and counted to determine how many were eaten. At the end of the experiments, fish survival was 100%, not differing between treatment groups (temperature, pregnancy and sex). Survival was also 100% in the stock tanks. The research was performed in accordance with the relevant ethical standards of the country where the research was performed and conforms to Directive 2010/63/EU. The experiments were approved by the Laboratory of Trophic Ecology of the Department of Environmental Biology (Sapienza University of Rome). The authors complied with the ARRIVE guidelines^64,65^.

### Data analysis

The FRAIR R package^66^ was used to model the guppies’ functional response for each single treatment (considering temperature and sex) as well as for pooled temperature data to highlight the effect of sex.

The T-statistic was used to compare the mean consumption of small and large prey at densities where they were not completely depleted (Experiment 1: mixed prey treatment). One-way ANOVAs and Tukey’s pairwise comparisons were performed to compare prey consumption between experiments with single-size and mixed-size prey and between temperatures (Experiment 1).

In accordance with Pritchard et al.^66^, the FRAIR procedure was applied in three steps. First, a logistic regression was used to determine functional response type, with Type-II being characterised by a negative first-order term and Type-III by a positive first-order term. Second, FRs were modelled using Maximum Likelihood Estimation (MLE^67^) with modifications proposed by Rogers^68^. Unlike the basic models^38^, which assume constant resource density throughout the experimental trials, Rogers’ equation makes it possible to model FRs even in cases where resources are not replaced^68,69^. MLE with Rogers’ modifications returns optimised values for attack rate (*a*) and handling time (*h*) with the relative *p*-values from the Z-statistics. In our study, the experimental time *T* was set to 1, which corresponded to 1 h. Lastly, Juliano’s difference method^70^ was used to test for differences in *a* and *h* values across experimental conditions (i.e. between temperatures, sexes and female pregnancy stages), and a non-parametric bootstrap procedure (*n* = 2000) was used to generate 95% Confidence Intervals (CIs) around the mean FR curves. For each FR, the maximum feeding rate (1/*h*T) was also calculated.

## Results

### Effect of sex and temperature on functional response

**Fig. 2:**
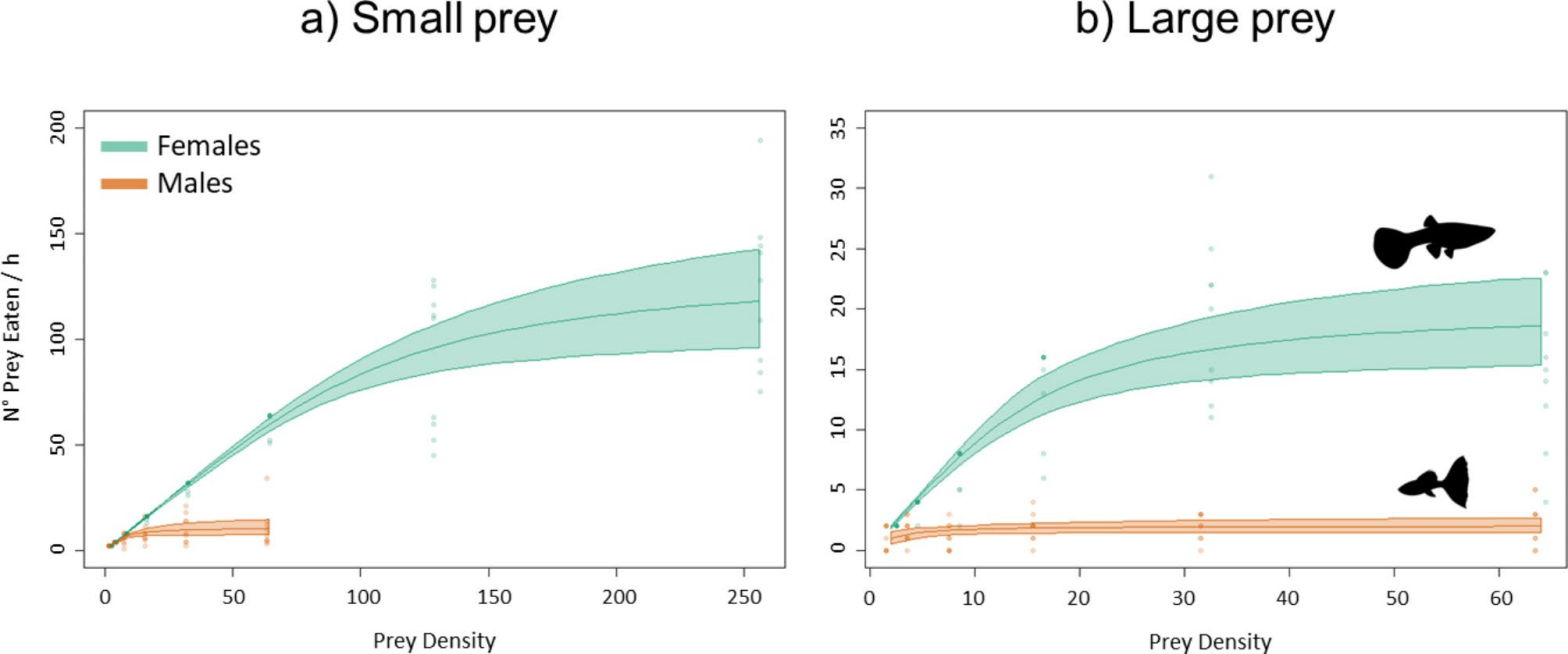
Functional response curves for female (teal line) and male (orange line) guppies fed small **(a)** and large **(b)** prey (pooled temperature data). The shaded area around the FR curves indicates the bootstrapped 95% confidence intervals. Please note the differences in the scale of the axes.

**Fig. 3:**
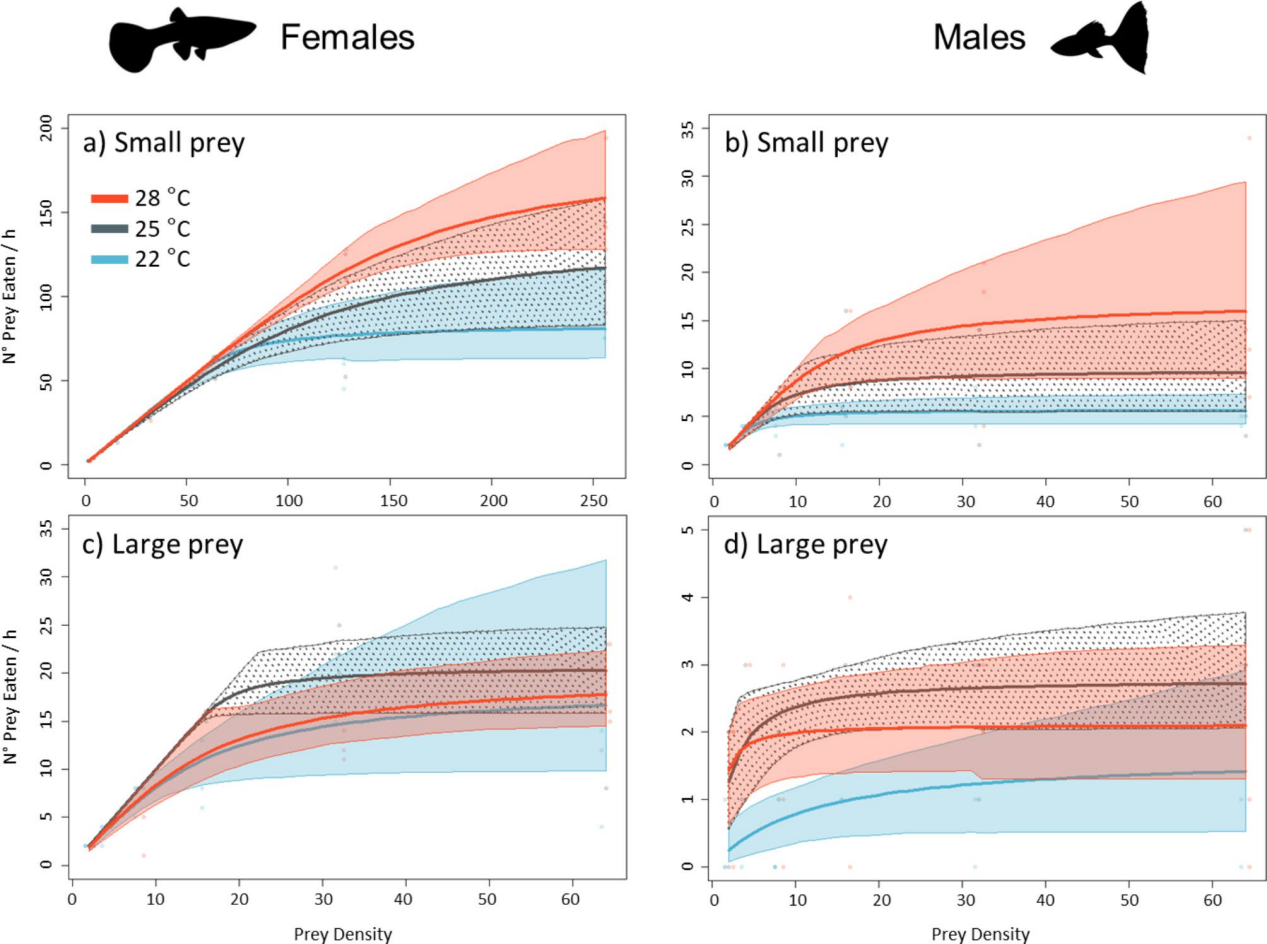
Functional response curves for female (left side) and male (right side) guppies fed small **(a, b)** and large **(c, d)** prey (*Chironomus aprilinus* larvae) at three temperatures. 22 °C: light blue line, 25 °C: grey line, 28 °C: red line. Shaded/dotted areas around the FR curves indicate the bootstrapped 95% confidence intervals. Please note the differences in the scale of the axes.

The logistic regression between the number of prey eaten by females and males and prey density had a significantly negative first-order term at all temperatures and prey sizes, indicating a Type-II Functional Response (Figs. 2 and 3; Tables 1 and 2).

**Table 1:**
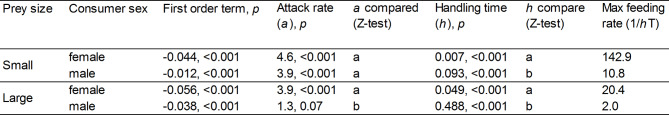
Functional response first order term, attack rate (*a*) and handling time (*h*) with associated *p*-values and maximum feeding rate, for female and male guppies fed small and large prey. Significant differences between male and female attack rates and handling times for each prey size are highlighted by different letters (Z-test, *p* always < 0.01).

**Table 2:**
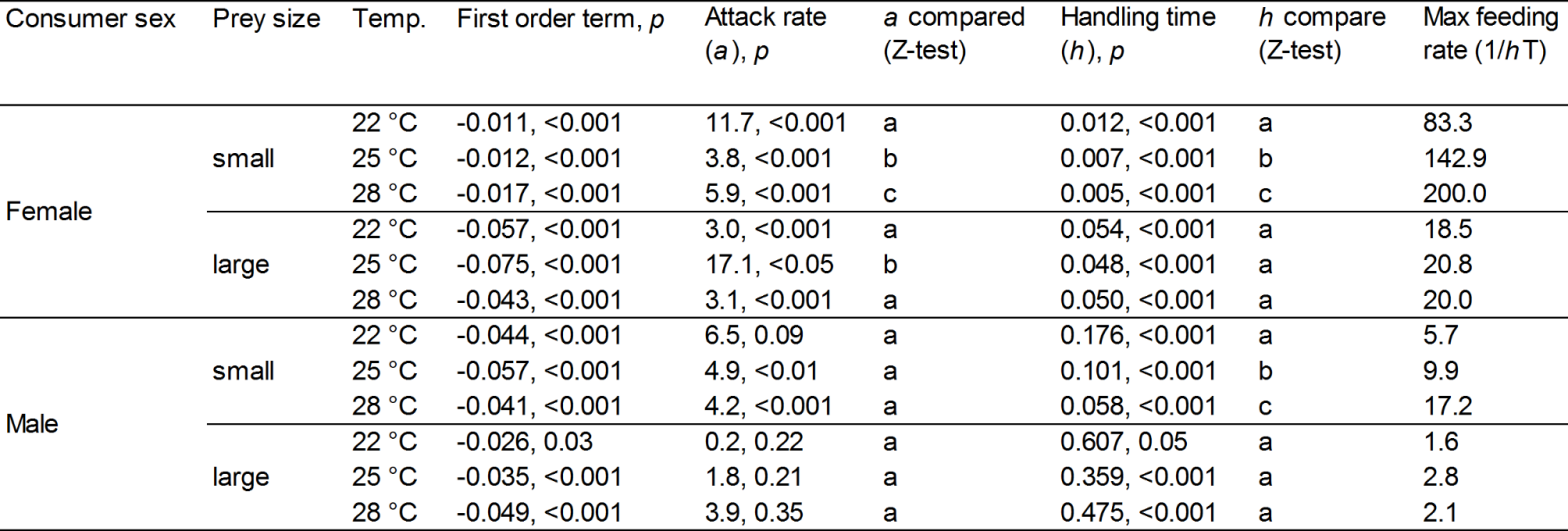
Functional response first order term, attack rate (*a*) and handling time (*h*) with associated *p*-values and maximum feeding rate, for the experimental conditions (combinations of predator sex, prey size and temperature) tested in experiment 1. For each sex and each prey size, significant differences between attack rates and handling times at the three temperatures are highlighted by different letters (Z-test, *p* always < 0.05). For further details, please refer to Sect. 2.2.

Overall, females had significantly higher FRs than males regardless of prey size (Fig. 2; Table 1). Prey handling time (*h*) was always significantly lower in females than males, while attack rate (*a*) was higher in females than males when feeding on large prey (Table 1).

When fed small prey, FRs and maximum feeding rate increased with temperature in both sexes (Fig. 3a, b; Table 2), while handling time (*h*) decreased. Females displayed remarkably higher attack rates (*a*) at the lowest temperature than at the other two temperatures.

When fed large prey, the FRs of both females and males did not differ between temperatures, nor did their constitutive parameters *a* and *h*, with the exception of attack rate (*a*) among females, which was higher at the intermediate temperature than the other two temperatures (Z test, *p* always ≥ 0.05) (Fig. 3c, d; Table 2).

### Effect of sex and temperature on prey-size selection

**Fig. 4:**
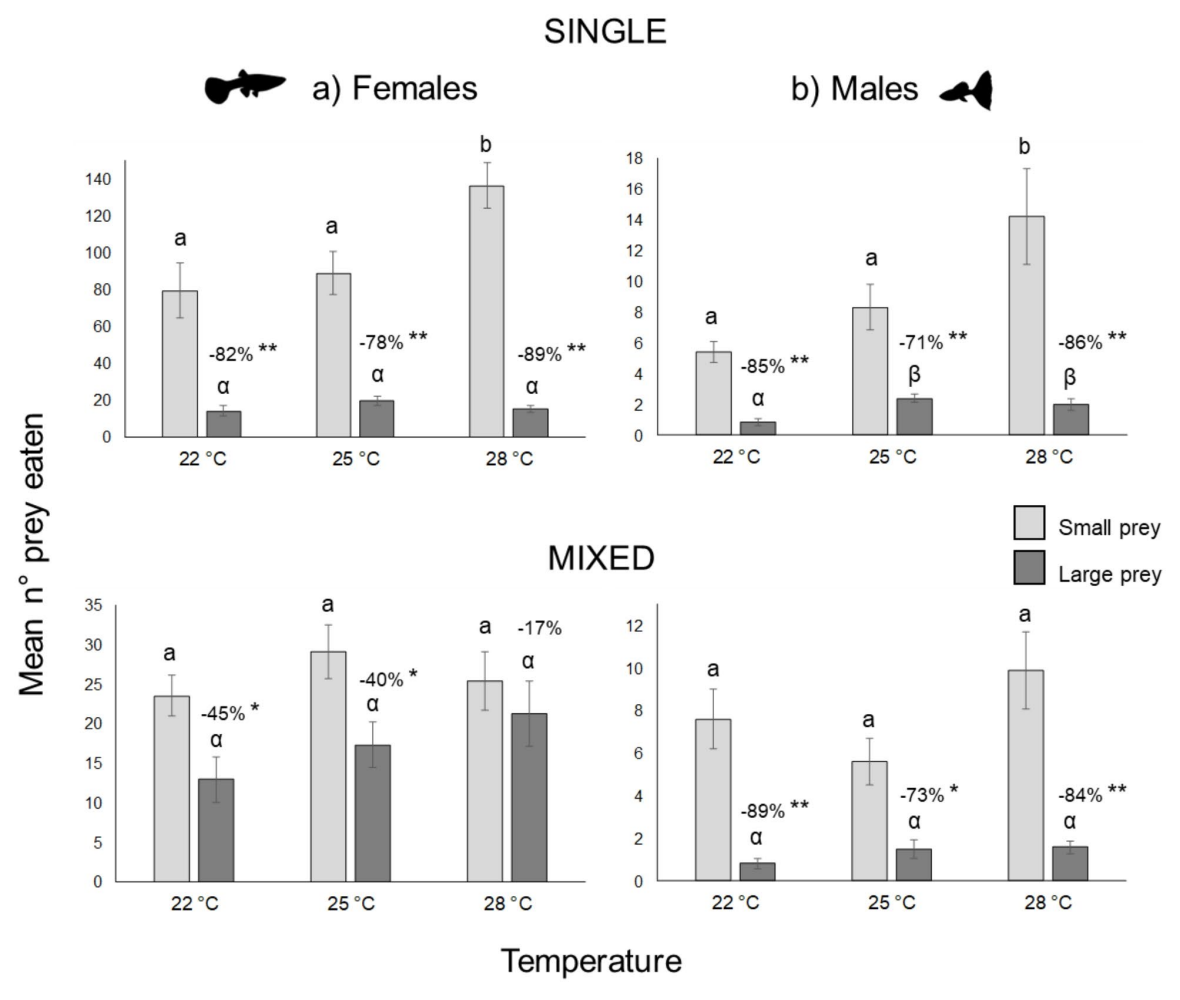
Numbers of small and large prey consumed by female **(a)** and male **(b)** guppies fed single-size prey (upper panels) and mixed-sized prey (lower panels) at three temperatures. Mean values ± standard errors are shown. The differences between small and large prey consumed by guppies at each temperature are explicitly indicated. Asterisks indicate significant differences between the mean consumption of small and large prey (T test, *: *p* < 0.05, **: *p* < 0.001). Please note the differences in the scale of the y-axes. Different letters indicate significant differences between small prey (Latin letters) and between large prey (Greek letters) at the three temperatures (one-way ANOVA and Tukey’s pairwise comparisons, *p* < 0.05).

In single prey-size experiments, females and males respectively consumed all small larvae present in the experimental tanks up to the 64 and 8 densities, and all large larvae up to the 16 and 2 densities. In both sexes the number of small prey items consumed by the guppies increased with temperature (Fig. 4 upper panels), varying from 79.2 ± 14.8 to 136.2 ± 12.4 in females and from 5.4 ± 0 0.6 to 14.2 ± 3.1 in males in an hour of feeding. Females consumed much fewer large prey items than small ones (78–89% less, Fig. 4a top panel), with a slightly higher value at the intermediate temperature.

In mixed prey-size experiments, neither small nor large prey were completely depleted by females at densities above 32. The number of small prey eaten by females was much lower (one-way ANOVA and Tukey’s pairwise comparisons, *p* always < 0.001, Fig. 4a), while the lower consumption of large prey with respect to the single prey-size experiments was less marked. The consumption of large prey increased more linearly with temperature (one-way ANOVA and Tukey’s pairwise comparisons, *p* always < 0.05, Fig. 4a), while that of small prey varied in accordance with a unimodal (hump shaped) model (Fig. 4a lower panel). Accordingly, the ratio of small to large prey in the females’ diet decreased with temperature.

No differences in the number of larvae consumed by males were observed between single and mixed prey-size experiments (one-way ANOVA n.s., Fig. 4b). Males consumed all larvae up to 4 individuals (2 small + 2 large). Above this number, more small than large prey were consumed (Fig. 4b lower panel).

**Fig. 5:**
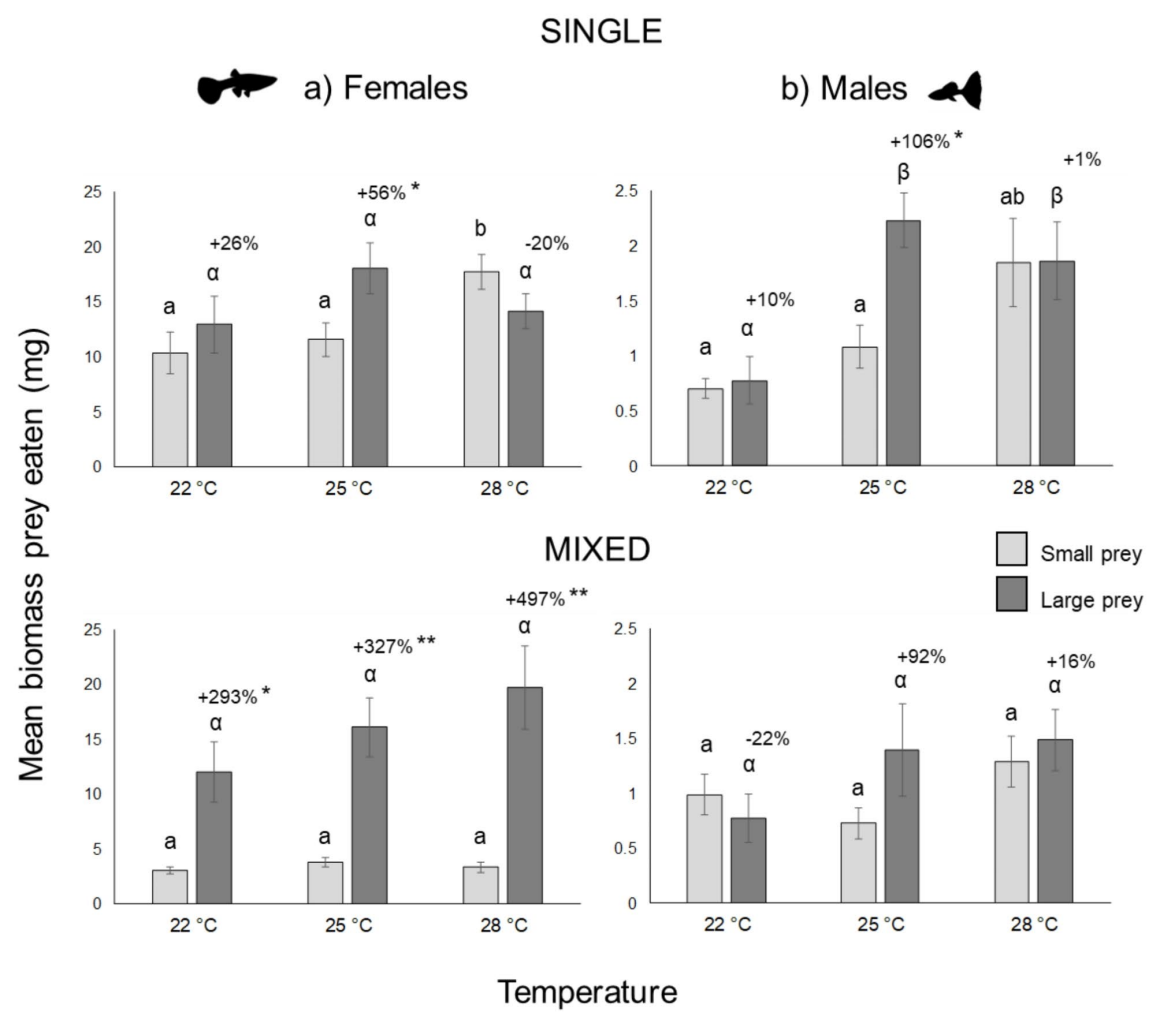
Biomass of small and large prey consumed by female **(a)** and male **(b)** guppies fed single-size (upper panels) and mixed-size prey (lower panels) at three temperatures. Mean values ± standard errors are shown. The differences between small and large prey consumed at each temperature are explicitly indicated. Asterisks indicate significant differences between the mean consumption of small and large prey (T test, *: *p* < 0.05, **: *p* < 0.001). Please note the differences in the scale of the y-axes. Different letters indicate significant differences between small prey (Latin letters) and between large prey (Greek letters) at three temperatures (one-way ANOVA and Tukey’s pairwise comparisons, *p* < 0.05).

In terms of biomass, the differences in prey consumption between single and mixed prey-size experiments were small (Fig. 5). The total biomass of prey consumed by females and males respectively averaged (± SE) 15.8 ± 1.4 mg and 1.7 ± 0.2 mg. For both sexes, biomass consumption was lowest when only small prey were offered at the lowest temperature and highest when all prey were offered at the highest temperature.

### Effect of pregnancy

**Fig. 6:**
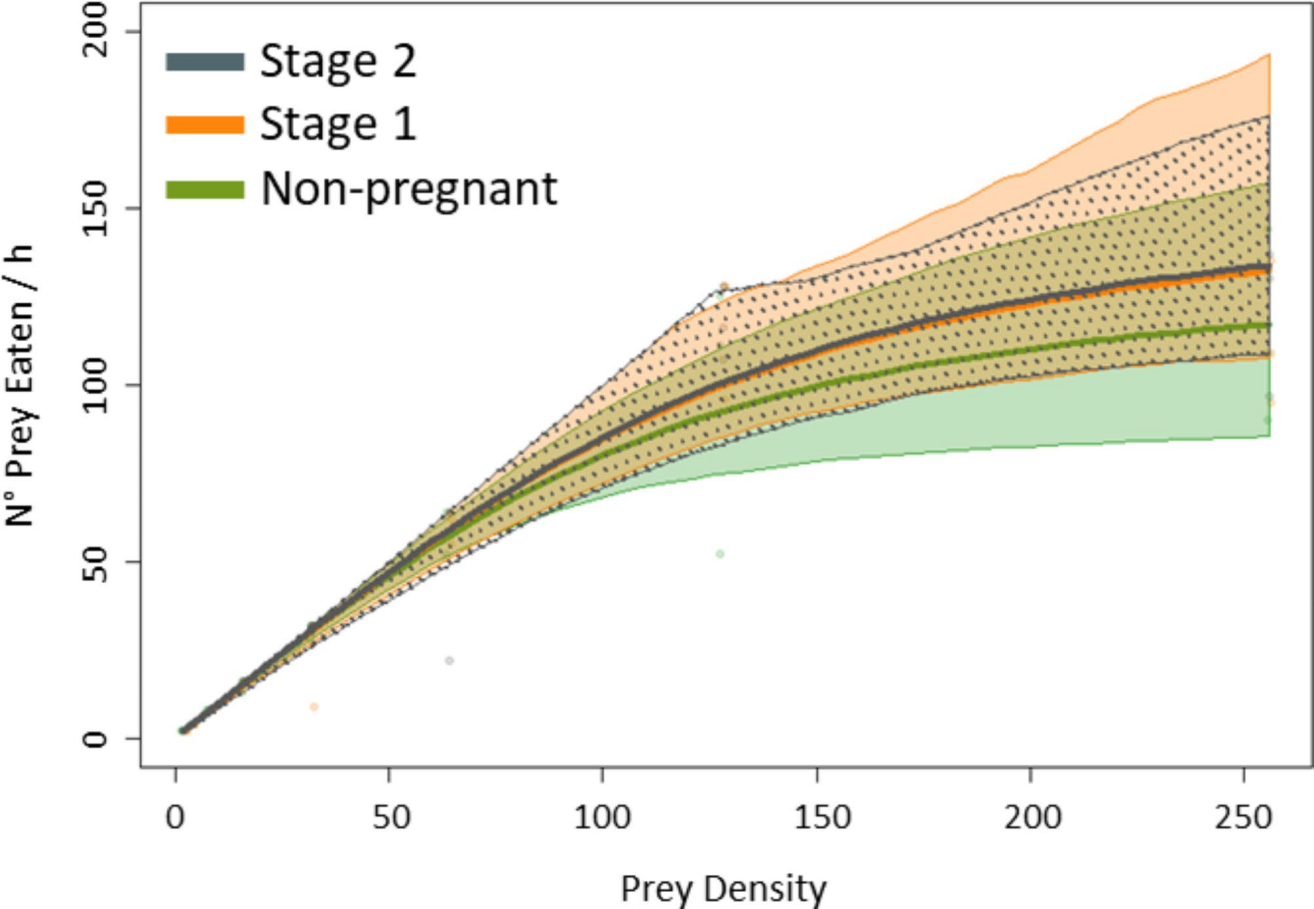
Functional response curves for non-pregnant and pregnant female guppies feeding at 25 °C on small prey (the only condition tested for pregnant females). Non-pregnant: green line; stage 1 pregnant (19 days of gestation): orange line; stage 2 pregnant (26 days of gestation): grey line. Shaded/dotted areas around the FR curves indicate the bootstrapped 95% confidence intervals. For further details, please refer to Sect. 2.2.

No significant differences in type II FRs were observed between non-pregnant and pregnant females (Fig. 6; Table 3). However, handling time *h* was significantly higher and maximum feeding rates lower in non-pregnant females (Table 3), while the two parameters did not differ between pregnancy stages. At the plateau, 88.7 (± 11.8 SE) prey were consumed by non-pregnant females, 96.9 (± 10.3 SE) by stage-1-pregnant females, and 119.2 (± 5.1 SE) by stage-2-pregnant females.

**Table 3:**
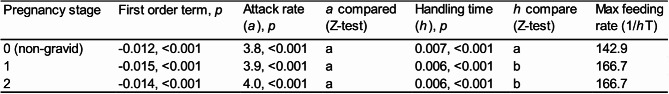
Functional response first order term, attack rate (*a*) and handling time (*h*), with associated *p*-values and maximum feeding rate, for female guppies at pregnancy stages 1 and 2, which correspond to 19 and 26 days of gestation respectively. Significant differences between attack rates and between handling times are highlighted by different letters (Z-test, *p* always < 0.05). For further details, please refer to Sect. 2.2.

The attack rate *a* did not differ between pregnant and non-pregnant females or between pregnancy stages (Table 3).

## Discussion

Functional Responses represent a measurable species trait with the potential to unify invasion ecology across taxa and habitats, with significant predictive ability^71^. However, they have rarely been compared between sexes and/or individual physiological states within single fish species populations, although they have been explored in some invertebrate taxa^72–74^. Neglecting sex differences, which are even more evident in sexually dimorphic species, has been highlighted as one of the main shortcomings of both laboratory and field studies of the response of species to climate change^32,33^.

In this laboratory study, functional responses, together with prey size selection, provided in-depth insight into the interactive effect of sex and warming on the feeding rates of the highly globally invasive guppy *Poecilia reticulata* used as a sexually dimorphic model species. Two-generation fish rearing prior to feeding trials allowed us to standardise specimens’ age and size and minimise their genetic variability, which can potentially introduce background noise and mask differences arising from experimental factors^75^.

Overall, the study found that temperature, sex and female physiological status (pregnancy) influence the fish’s feeding activities and maximum feeding rates (i.e. the magnitude of the functional response curve). Specifically, the results highlight four main points: (1) guppies’ functional responses to increasing prey density differed between sexes; (2) increased temperature triggered increased functional responses in both sexes; (3) increased temperature influenced females’ prey size selection; (4) pregnancy reduced prey handling time.

Based on the maximum feeding rate, the observed FRs were about 10 times higher in females than in males. These differences may be explained with reference to fish body size, as reported for wild guppies^76^, and/or physiological processes that differentiate females from males, and this may apply especially to species with pronounced sexual dimorphism, including most poecilids^77^, as well as fish families belonging to the most widespread fish groups (e.g. Characiformes, Cypriniformes, Siluriformes, Gymnotiformes, Perciformes and Cyprinodontiformes)^13,78^. Similar but less evident differences in FRs between males and females have also been found in sexually size-dimorphic birds^79^.

In order to enable FR to take account of metabolism, Basset et al.^80^ proposed an allometric model linking ingestion and metabolic rates with body size and resource availability. This model explained the body size dependence of the half-saturation coefficient of FR (i.e. the resource density at which the feeding rate reaches half the maximum) in benthic detritivores. Our results show that FRs increased with temperature in both fish sexes. At the highest temperature, they were more than double those at the lowest temperature in females and more than triple in males. This difference can be at least partly explained by the allometric scaling of physiological traits with body size. Metabolic rate, which is affected by temperature especially in ectotherms^29,81,82^, does not increase linearly with body size^81,83^, in fish increasing with body weight (W) at a rate of about W^0.8^^83,84^. Lindmark et al.^85^ found that maximum consumption in fish increases even more slowly with body mass than metabolism, and is hump-shaped when correlated with temperature. According to the authors, unimodal relationships between consumption rate and temperature indicate decreasing optimal temperature for net energy gain and individual growth with increasing body size. This makes the impact of warming on fish populations unpredictable if individual size variability is not adequately accounted for.

By reducing the predator’s prey handling time, increasing temperature can increase the maximum feeding rates and thus the predatory impacts of females and males on prey and food webs^45,50,52^. In FR models, handling time comprises both active behaviours (handling, which is related to capture and consumption) and physiological processes (digestion). Sentis and colleagues^45^ showed that in a ladybird–aphid system, the digestion rate increases exponentially with warming, whereas the relationship between handling rate and temperature is hump shaped. Handling and digestion rates, which together determined the maximum feeding rate, were not individually investigated in our study. However, we observed a much higher maximum number of small prey eaten by females within the experimental time in the single-size experiments than in the mixed ones, while the number of large prey eaten by females was roughly the same. This indicates that prey handling was not an issue but satiety played a crucial role in determining the maximum feeding rate. This is consistent with the literature, which suggests that in fish, FRs are influenced by prey size. More specifically, it has been documented that satiety results from the predator consuming the maximum volume of prey, rather than number, that physiological constraints allow it to ingest^86^. In addition, while in single prey-size experiments the number of small prey eaten by the guppies increased monotonically with temperature in the applied range, the number of large prey was higher at the intermediate temperature in both sexes, confirming the role of satiety due to the higher prey biomass. In experiments with mixed-sized prey, the ratio of small to large prey in the diet of females decreased with temperature due to the increasing biomass of large prey ingested.

Furthermore, reproduction-related physiological changes may trigger major feeding responses in females. Pregnancy can lead to increased energy consumption^87^ and thus a higher impact on available prey than is seen in non-pregnant conditions. Females have high energy demands for egg production, which can be copious in terms of number and biomass, especially in larger individuals (in some cases several million eggs, in others accounting for up to 40% of total body weight^88^). The energy and protein requirements for egg production per unit of biomass have been found to be greater than depositing new tissue for growth^89^. Increased energy requirements can also result from pregnancy-related physiological changes such as increased work for the cardiac and branchial pump, which both lead to increased oxygen consumption^87^. Our study showed that females had lower handling time than males and pregnant females even lower than non-pregnant ones. This suggests that a higher maximum feeding rates in females, whether pregnant or not, could increase the overall impact of non-native dimorphic fish on native prey. Sex ratios in natural fish populations are often female-skewed due to factors including environmental sex determination, differential sensitivity to temperature, growth rates and predation^90–93^. Indeed, it has been found that males have shorter life spans^94^ and are more sensitive to higher temperatures^90^, more subject to predation^95,96^ and less tolerant of low resource levels^95^. This implies that several ongoing global changes affecting freshwater ecosystems, such as global warming, the introduction of non-native predators and habitat simplification, could favour female-dominated populations. Differences in prey consumption rates between sexes could lead to numerically similar populations differing in their overall predatory impacts on invaded food webs due to dissimilar sex ratios.

Female-biased sex ratios have often been found in wild populations of guppies^94,95,97^ and other mosquitofish, inducing stronger pelagic trophic cascades than male-biased populations^98^. Female-biased populations have also been found in other fish groups^99–102^. However, community ecology models tend to view the sex of organisms as irrelevant, treating males and females as metabolically equivalent, and studies of invasive species typically consider average trophic interactions, with females and males pooled together^33,103–105^.

When sexual dimorphism exists, size-specific prey availability can also play an important role in determining the foraging success of the invasive fish predator and its impact on food webs. Sexual dimorphism allows males and females to exploit different food resources, thus increasing their collective ability to find food^106^. Their success also depends on their ability to modify their diet when availability does not match their energy need^106^. The use of differently sized conspecific prey in the experiments mitigated the potential confounding effects of species-specific factors modifying prey selection (e.g. body shape, composition in terms of tissues and nutritional quality). A correlation between body size and trophic level frequently exists in animals^37^, and this can also apply to conspecific larvae of several chironomid species which are known to feed on basal food sources when smaller and on animal prey when larger^37,107^. Consistent with our results, this suggests that sexually dimorphic species, such as *Poecilia reticulata*, can affect various trophic levels in invaded food webs depending on their sex ratio, prey size availability and temperature.

In addition, female guppies and other tropical fish may produce eggs more frequently when they are at their optimal temperature^108–110^. Many of these fish species are widespread in sub-tropical and temperate regions, where they represent a threat to freshwater biodiversity^24,25,57^. Due to climate change, they may experience thermal conditions that are closer to their optima outside their original geographical range. Thus, together with the expected increase in offspring and feeding rates, a greater frequency of pregnancy may worsen the impact of females on their prey populations in invaded communities.

In conclusion, this study allowed us to gain insight into the potential consequences of rising temperature due to climate change for feeding interactions between tropical invasive fish and their prey.

The results highlight the need to consider variations in sex ratios and their responses to temperature in order to accurately identify the role of invasive species in invaded communities. Intraspecific feeding patterns determine how species interact within food webs and can vary in response to environmental changes^41,111,112^. They are thus relevant to predicting the predatory impact of invasive fish on communities in a warming world. When such invasive populations exist, ignoring sex differences in prey consumption can lead to assessments of trophic interactions that are not fully representative of the invaded food webs. This in turn can make management measures against biological invasion less effective.

## Data Availability

Raw data used in the manuscript that are not already provided are available from the corresponding author on reasonable request.
